# Medical fitness to drive, emergency service vehicles and crash risk

**DOI:** 10.1007/s11845-023-03301-0

**Published:** 2023-02-08

**Authors:** Donna Noonan, Margaret Ryan, Declan Whelan, Desmond O’Neill

**Affiliations:** 1 National Office for Traffic Medicine, Royal College of Physicians of Ireland, Dublin, Ireland; 2 Centre for Ageing, Neuroscience and the Humanities, Trinity Centre for Health Sciences, Tallaght University Hospital, Dublin, D24 NR0A Ireland

**Keywords:** Automobile driving, Crashes, Emergency responders, Risk assessment, Traffic

## Abstract

**Background:**

Emergency service vehicle (ESV) drivers are an important part of the health, fire and police services. ESV driving is associated with increased crash risk, but little guidance exists in the literature on relevant medical conditions among drivers and their potential for adding to higher crash risks.

**Aims:**

We undertook a narrative review to examine the role of medical and other conditions in crash risk of ESV drivers.

**Method:**

A literature search was conducted using the ScienceDirect and Transport Research International Documentation (TRID) databases. There was no time frame for the search, and results were restricted to review and research articles.

**Results:**

Of 570 papers identified, 13 remained after screening and full-text review. A range of factors have been shown to have an impact on increased crash risk, including the nature of the task, physical features of the equipment, training, experience, environmental conditions and secondary tasks. There was scant information on medical conditions other than alcohol use disorders.

**Conclusions:**

Given issues of speed, vehicle and environment, it would seem prudent to mandate levels of medical fitness to drive similar to and sometimes exceeding (i.e. colour blindness for traffic signals and alerts, hearing impairment as first responders) those for group 2 drivers with extra stipulations relating to specific service needs such as enhanced visual (such as colour blindness and contrast sensitivity) and auditory function. Further research is needed on the prevalence and emergence of relevant medical conditions among ESV drivers, with due consideration of their application to the driving tasks in each service.

## Introduction and aim

Emergency service vehicle (ESV) or blue light drivers are an important part of the public health, fire and police services. In many countries, including Ireland, members of the police, ambulance services and fire brigades are not bound by road traffic legislation (such as speed limits and adherence to traffic signals) when drivingin the performance of their duties where such use does not endanger the safety of road users.

They are at the front line in responding to accidents and emergency situations. The unique circumstances in which blue light driving occurs are challenging and arguably heighten the crash risk for blue light drivers as compared with driving in personal and other occupational situations [1]. It has been well documented that driving poses a ‘hidden risk’ to ESV personnel [2], and drivers of ambulances, police cars and fire engines are at risk of serious injury and death after a crash [3]. In Finland, occupational disability risk for paramedics is 2.4% compared with the national average of 1.7% [4]. Statistics from the USA indicate that occupational fatalities amongst ESV drivers are 2.5 times higher than the national average with almost three-quarters of these caused by traffic-related accidents [2]. Figures from Australia suggest ambulance personnel have an occupational fatality rate six times the national average, with 85% of fatalities attributed to crashes [5].

The additional crash risk associated with ESV driving highlights the importance of assessing medical fitness to drive and ESV drivers. A background to such concerns is that of the fitness of personnel in emergency services—in international studies, police personnel are more likely to have a high prevalence of traditional risk factors, including hypertension, hyperlipidaemia, metabolic syndrome, cigarette smoking, and a sedentary lifestyle [6], a pattern seen among other ESV personnel [7]. Of potential importance is also the recognition in the international literature of high levels of alcohol use among police personnel [8]: alcohol use disorders were a key factor in early retirement of ambulance personnel, at a level significantly higher than other healthcare personnel [9].

In the UK, a recommendation has been made by the Faculty of Occupational Health that guidelines for medical fitness to drive should be based on those for group 2 drivers, although further defined as a group 2-minus with allowance for clinical discretion after expert evaluation of the driver, but also noting the presence of certain groups of blue-light drivers in the UK who are not permitted to disregard traffic regulations in an emergency, such as mountain rescue teams and His Majesty’s Coastguard [10]. This guideline also notes a recommendation from the DVLA Panel on Diabetes and Driving that drivers treated with insulin should not undertake blue-light driving.

### Emergency Services Driving Standard (ESDS)

In Ireland, a new voluntary training system for ESV drivers, the Emergency Services Driving Standard (ESDS), was introduced in 2014 by the Road Safety Authority (RSA) as part of the Irish Government’s Road Safety Strategy 2013–2020. It was developed in response to ESV professionals outlining the need for training and management in emergency response driving [11]. As of 2019, 2384 ESV drivers have been certified to the ESDS [11]. The ESDS training programme comprises of three levels and covers the skills, knowledge and behaviours that full-time, part-time and volunteer ESV drivers should demonstrate when they are required to drive emergency vehicles [12]. Although the syllabus refers briefly to the importance of being fit to drive in terms of fatigue, and alcohol and drug use, there is no specific reference to medical fitness to drive [12].

### Medical fitness to drive (MFTD)

In order to drive safely, a person must be medically fit and able to do so. Certain medical conditions may hinder a person’s ability to drive safely. In Ireland, the Sláinte agus Tiomáint medical fitness to drive guidelines outline medical standards to guide the health assessments of drivers for licensing drivers in Ireland. Separate standards are applicable for group 1 (car, motorcycle, tractor) and group 2 (truck or bus). Group 1 drivers are generally driving for personal reasons, whereas group 2 drivers are normally professional drivers. As detailed in the guidelines, group 2 drivers often have extra conditions regarding medical fitness to drive as they are at elevated risk whilst on the roads [13]. Group 2 drivers tend to spend more time on the road than group 1 drivers which puts them at greater risk of a crash. Additionally, the vehicles driven by group 2 drivers pose a greater crash risk due to their increased height and weight and the risk of chemical spillage and damage to property [13]. ESV drivers in the health services in Ireland who are likely to drive with ‘blue lights’ must comply with the group 2 medical standard, or in some circumstances, with a higher medical standard has been applied to these drivers [14]. Firefighter services are delivered by county councils, and while medical standards for recruitment disbar relevant conditions such as epilepsy, all types of diabetes mellitus and alcohol dependence (Medical Requirements for Recruit Fire-Fighters), there is little clear guidance on emergent medical conditions. For the police, there are three levels of driving expertise, CBD 1–3: CBD permits exceeding the speed limit and using lights and siren. There is currently no reference of medical fitness to drive standards for ESV drivers in the Irish medical fitness to drive guidelines.

The purpose of this scoping review is to survey the international literature to identify specific crash risk factors associated with ESV drivers. Scoping reviews can clarify concepts in the literature and define gaps in knowledge. Unlike systematic reviews, scoping reviews do not aim to produce a critically appraised and synthesised result or answer to a particular question, rather aim to provide an overview or map of the evidence. In this case, the aim is to highlight gaps in the literature pertaining to ESV drivers and crash risk. This will act as a basis for proposing additions to medical fitness to drive for ESVs in the *Sláinte agus Tiomáint* medical fitness to drive guidelines.

## Method

### Literature search

A literature search was conducted using the ScienceDirect and TRID databases. There was no time frame for the search, and results were restricted to review and research articles. In the initial search, the search term ‘blue light vehicle’ and ‘crash risk’ and ‘driver fitness’ was used. This failed to yield any relevant articles as the term *blue light* is largely absent from the literature. Therefore, ‘blue light’ was replaced with ‘emergency service vehicle’ which resulted in more relevant results. Separate searches including the terms ‘ambulance’, ‘fire truck or fire engine’ and ‘police car’ were used to identify articles specifying these drivers in particular. A full list of search terms can be found in Table 1. A Google Search was also conducted in order to capture relevant ‘grey literature’ including manuals and reports.

### Study selection

All search results were uploaded to Endnote citation software. The results were screened for duplicates, and then, titles were screened for relevance. The abstracts of relevant titles were screened, and articles for full-text review were selected. Articles which were included in the final selection had to discuss at least one type of emergency vehicle driver and one aspect of crash risk for the ESV driver.

## Results

The literature available regarding emergency service vehicles and crash risk is relatively limited with the majority focusing on research conducted in Australia and the USA. However, in recent years, there has been a small resurgence in the material written on crash risk and ESV driving with some focus on the European context.

**Fig. 1:**
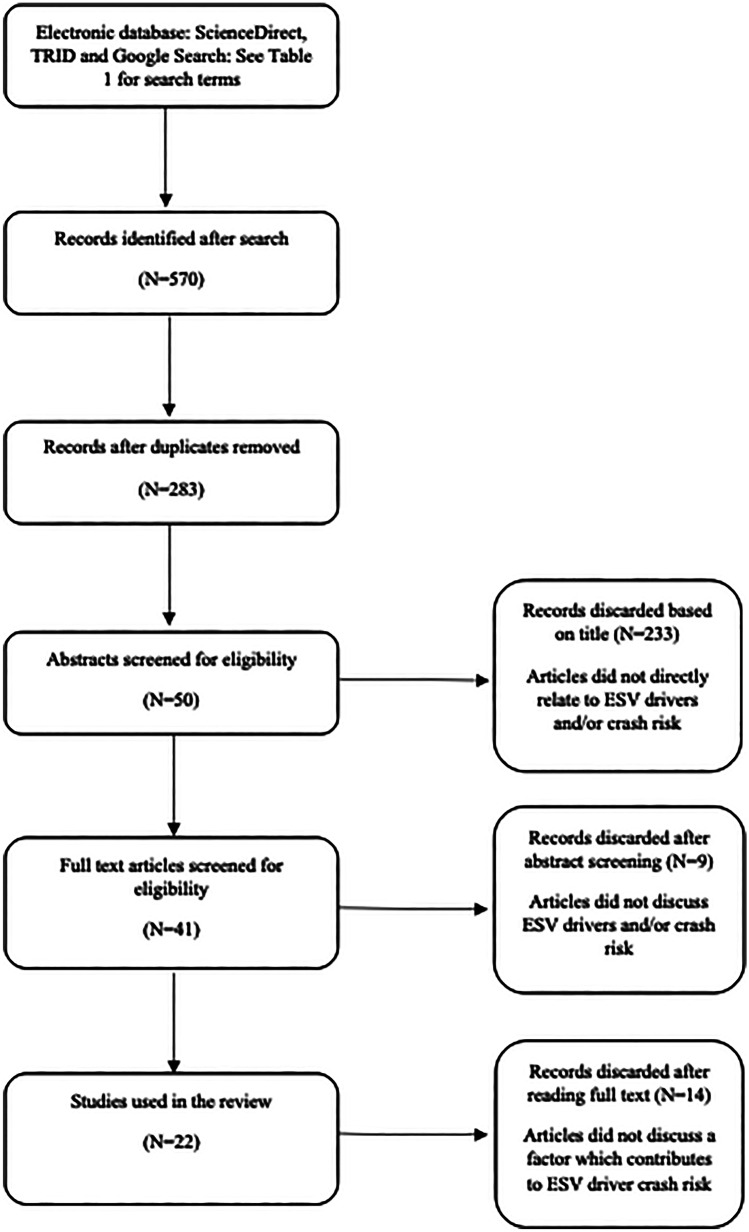
Search strategy for literature review

Several factors related to ESV driving and crash risk are discussed in the literature. They have been divided into four main sections for the purposes of this review: personal factors, the emergency vehicle driving task, the emergency vehicle and the environment. Figure 1 documents the article selection process.

### Personal factors

The personal factors of ESV drivers discussed in the literature regarding crash risk include driver experience and driver behaviour. The literature on driving in the general population indicates that driver age (youthfulness) and inexperience are key contributors to crash risk [15]. This is consistent with the results of the majority of studies that investigated crash risk and experience in ESV drivers. An early study by Custalow and Gravitz (3) which examined retrospective crash data of ESV drivers in an 8-year period from 1989 to 1997 showed that the majority of vehicle crashes involved drivers with less than 3 years of ESV driving experience. Similar results were found more recently where 42% of ESV drivers involved in crashes were aged between 18 and 29 [16]. This trend was reflected in a Finnish study which surveyed paramedics and focused on what risk factors they recognised when performing emergency driving [4]. It highlighted a lack of specific ESV driver training: for many young paramedics, it is their first time driving a larger vehicle when dispatched on a call-out. Although Custalow and Grawitz suggest that ESV drivers become more cautious over time and with gained experience [3], it has also been suggested by the United States Fire Administration that as drivers gain more experience, they become overconfident which leads to greater crash risk [17]. This suggestion has also been made by Abeldwanis that ESV drivers over the age of 50 could be more prone to crash than their younger counterparts [18].

A salient point in the literature regarding ESV drivers and experience is the question of voluntary ESV drivers. They are a particular group of ESV drivers in that they engage in ESV driving as a voluntary activity and not as a primary occupation. The irregular nature of volunteer driving coupled with the fact that volunteer ESV drivers are potentially driving vehicles they are not very familiar with could lead to increased crash risk [1]. Furthermore, voluntary ESV drivers have generally strong altruistic motivations to engage in ESV driving and may not disclose if they are unfit to undertake the ESV driving task [1]. Volunteer ESV drivers are under-represented in the already sparse literature on ESV drivers and crash risk and warrant further study.

Driver behaviour can also be a lead cause of crash risk in ESVs. Individuals in high-risk situations do not always react like they would under normal circumstances [19]. A review concluded that engaging in excessive speeding, harsh braking and hard cornering were behaviours associated with crash risk in the fire service [20]. As posited by Abdelwanis (18) regarding older ESV drivers, overconfident driving behaviour has also been highlighted as a possible risk factor in ESV driving [21]. Overestimation of driving skills, particularly in good driving conditions, was one of the risk factors highlighted by Finnish paramedics when engaging in emergency response driving [4]. Furthermore, repeated risky driving behaviours of ESV drivers are significant determinants of emergency vehicle incidents [2]. This is supported by research indicating that ESV drivers who had already been involved in a crash were likely to be involved in another [22]. The tendency for repeat crash risk is worrying, and preventive measures are needed to combat this trend.

### The emergency driving task

The driving task performed by ESV drivers is arguably more complex than personal or other professional driving. ESV driving is undertaken in high-pressure, time-sensitive situations with significant stress attached. Additionally, ESV drivers commonly work in a shift pattern which increases the likelihood of fatigue and drowsy driving [23]. ESV drivers are often required to engage in emergency or ‘lights and sirens’ driving in an emergent situation. Thus, the ESV driver may engage in speeding and red-light running to attend the scene of an emergency.

The majority of ESV crashes occur during ‘lights and sirens’ response [24]. The accident rate for ESV drivers in Dublin was three times higher when attending the scene of an emergency compared with leaving it [25], and five times higher while using the ESVs were in lights and sirens mode in Denver [3]. This is likely linked with the fact that ESV drivers often engage in speeding while in lights and sirens mode. Speeding decreases the time available to either manoeuvre or stop to avoid a crash [26].

However, some contradictory evidence also exists in the literature. Although the majority of crashes occur during ‘lights and sirens’ response, one study showed that the risk of injury and death is higher when an ambulance is driving in regular mode [24]. One commentator suggests that ESV drivers are on high alert during emergency response and heightened concentration may lead to improved vehicle handling and crash avoidance [20]. Furthermore, a quasi-induced exposure study on ESV crash statistics in Iowa in the USA showed that although police vehicles were 1.8 times more likely to crash in lights and sirens mode, ambulance and fire engines were not more likely to crash [27]. This could be related to the fact that police vehicles are smaller and can reach higher speeds, so crash risk increases [27].

The complexity of the driving task is coupled with the secondary task demands of ESV driving. While driving, ESV drivers may also need to answer a radio or speak with a colleague or other vehicle occupant. The majority of the literature discussing distraction and ESV driving focusses on crash risk and police vehicle drivers. A controlled laboratory experiment using a simulator concluded that police officers were more likely to have longer braking reaction times and have greater lane deviation while using a mobile data computer [28]. Similar results indicated that 69% of 48 police vehicle crashes experienced by the Austin Texas Police Department were attributed to interacting with a secondary device such as a phone or mobile computer [29]. One of the reasons proposed for the prevalence of crash risk amongst police vehicle drivers is that they usually operate with a smaller crew compared to fire engine or ambulance personnel, thus increasing the burden of secondary tasks [29]. More education and training in ‘lights and sirens’ driving and advancements in technology may reduce potential crash risk.

### Vehicle design

The nature of emergency vehicles is another consideration for crash risk. Ambulances and fire engines in particular are large, cumbersome vehicles. Due to their size and weight, ambulances are more difficult to stop than traditional vehicles, particularly on slick road surfaces [3]. More time is required for acceleration and deceleration than regular vehicles; therefore, adequate training is essential to mitigate crash risk [17, 26].

In the case of ambulances, particular consideration should be given to ambulance design and function. Patient care during transport is an inherent feature of driving an ambulance. However, this unavoidable element of the job puts ambulance personnel at a higher risk of crash [30], albeit that the most serious and fatal injuries occur in the rear compartment of an ambulance [31]. Ambulance design is not directed to occupant protection, and elements such as sharp edges on cabinets, poorly designed seatbelts, and the risk of projectiles put personnel and other occupants at higher risk of injury in a crash [30, 31]. Although concerns about the design of ambulances have been documented in earlier studies and improved design recommended [22], more recent work shows that newer ambulances are not safer for ESV personnel and occupants [30]. This demonstrates the need for further investigation into the design of ambulances to mitigate the risk of injury and fatalities in the event of a crash.

### Environmental conditions

Environmental conditions such as road design and weather may also attribute to crash risk in ESV drivers. Driving is a complex task, and added complexity on roadways such as intersections and traffic signals may increase crash risk. Ambulances are more likely to crash at intersections and traffic signals than other vehicles of a similar size [32]: occurrence of collisions at intersections was highly predictive of injury or fatality [3]. This increased crash rate could be linked to the increased stopping time necessary for larger vehicles such as ambulances and fire engines and a lack of local road knowledge [26].

Furthermore, ESV driving is an essential activity that has to be performed despite weather conditions. Adverse weather and poor road conditions are a significant risk factor for ESV drivers [3], consistent with findings in Northern Finland which showed the majority of ambulance crashes occurred during heavy snow [4]. Another consideration related to weather conditions is glare from the sun. Although Gormley and Walsh highlighted that glare from the sun was a worry for ESV drivers in the Dublin fire brigade [25], no further discussion of glare and ESV driver crash risk was found in the international literature. This could warrant further enquiry.

## Conclusions and recommendations

ESV drivers display higher levels of crashes, for which a range of factors have been shown to have an impact, including the nature of the task, the physical features of the equipment, training, experience, environmental conditions and secondary tasks. Less obvious in the literature surveyed was any linkage between recognized susceptibility to a range of relevant medical conditions among these drivers and their potential for adding to these higher crash risks.

Although the current international literature available on ESV drivers is relatively sparse, it shows a distinct trend towards increased crash risk and ESV driving. It is therefore important to identify where improvements can be made to make ESV driving safer for drivers, passengers, other occupants and road users.

Personal factors such as experience and behaviour could increase crash risk. Some inconsistencies exist in the literature regarding the effect of experience with some suggesting crash risk decreases with experience [16], while others indicated the opposite [18]. Continued training and education during the working lifespan of ESV drivers could ensure their skills stay sharp and current, mitigating crash risk. Increased training and education on how to drive in difficult weather conditions and negotiating complex road design features could also help to minimise crash risk.

Although earlier studies indicated that improvements in ESV vehicle design could decrease crash and fatality risk, more recent work has shown that newer vehicles are not significantly safer [30]. Further research and advancements in ergonomic design could also reduce crash risk for ESV drivers.

This scoping review highlighted the lack of research conducted to date regarding volunteer ESV drivers. They are a unique group of ESV drivers and may only perform ESV driving tasks on an extremely irregular basis and in unfamiliar vehicles. Further investigation is warranted to investigate if their crash risk is elevated compared to career ESV drivers.

ESV drivers provide an extremely important and essential service to communities the world over. As demonstrated by the international literature, ESV driving is a complex task that exposes ESV drivers to increased crash risk. Therefore, it is pertinent to explore provisions for fitness to drive and ESV drivers in the *Sláinte agus Tiomáint* medical fitness to drive guidelines to ensure safer roads for ESV drivers, passengers and other occupants, as well as other road users.

As the average age of ESV drivers is less than that of the driving population at large, it would seem important that medical guidelines for ESV drivers should prioritise medical conditions that are most prevalent between the young and middle stages of adulthood. These would include alcohol and substance use disorders, mental health, diabetes mellitus, vision, and medications. Due attention should be given at recruitment for the presence of neurodevelopmental conditions such as attention deficit and hyperactivity disorder and autism. However, between increasing focus on longer working lives, as well as concerns over the pattern of health behaviours of ESV drivers [6, 7], there will also need to be awareness of cardiovascular and cerebrovascular disorders as well as including early onset dementia.

Given the issues of speed, vehicle and environment, it would seem prudent to mandate levels of medical fitness to drive similar to and sometimes exceeding (i.e. colour blindness for traffic signals and alerts, hearing impairment as first responders) those for group 2 drivers. This recommendation is consistent with some elements of the UK Faculty of Occupational Medicine but supersedes in that we would recommend group 2 plus, i.e. group 2 with extra stipulations relating to specific service needs such as enhanced visual (such as colour blindness and contrast sensitivity) and auditory function. In the Irish context, this recommendation is a prudent measure given the current heterogeneity of occupational health services among ESV service providers but could be reviewed in due course. Further research is needed on the prevalence and emergence of relevant medical conditions among ESV drivers, with due consideration of their application to the driving tasks in each service.

**Table 1:** Search terms used in the literature search

Search number	Search term
1	Emergency service vehicle AND crash risk AND driver fitness
2	Fire engine OR fire truck AND crash risk AND driver fitness
3	Ambulance AND crash risk AND driver fitness
4	Police car AND crash risk AND driver fitness
5	Voluntary AND emergency service vehicles AND crash risk AND driver fitness

## References

[1] Muir C, Newnam S, Newstead S, Boustras G (2020). Challenges for safety intervention in emergency vehicle fleets: a case study. Saf Sci.

[2] Maguire BJ, Hunting KL, Smith GS, Levick NR (2002). Occupational fatalities in emergency medical services: a hidden crisis. Ann Emerg Med.

[3] Custalow CB, Gravitz CS (2004). Emergency medical vehicle collisions and potential for preventive intervention Prehospital Emergency Care.

[4] Koski A, Sumanen H (2019). The risk factors Finnish paramedics recognize when performing emergency response driving. Accid Anal Prev.

[5] Scott-Parker B, Curran M, Rune K (2018). Situation awareness in young novice ambulance drivers: so much more than driving. Saf Sci.

[6] Zimmerman FH (2012). Cardiovascular disease and risk factors in law enforcement personnel: a comprehensive review. Cardiol Rev.

[7] Kales S, Tsismenakis A, Zhang C, Soteriades E (2008). Blood pressure in firefighters, police officers, and other emergency responders. Am J Hypertens.

[8] Violanti JM, Slaven JE, Charles LE (2011). Police and alcohol use: a descriptive analysis and associations with stress outcomes. Am J Crim Just.

[9] Rodgers LM (1998) A five year study comparing early retirements on medical grounds in ambulance personnel with those in other groups of health service staff. Part II: Causes of retirements. Occup Med (Lond) 48(2):119–32 10.1093/occmed/48.2.119 9614771

[10] Faculty of Occupational H (2013) Guidelines for assessing fitness for blue light drivers. London: RCP London

[11] An Garda S (2019) Almost 2,400 Emergency service drivers certified to higher driving standard since 2014

[12] Rsa (2018) Syllabus: emergency services driving standard. RSA

[13] Rsa (2022) Sláinte agus Tiomáint: medical fitness to drive guidelines. Mayo: RSA. Report No.: 10 ed

[14] Hse (2018) HSE safe driving for work policy 2018. Dublin: HSE

[15] Christie R (2001) The effectiveness of driver training as a road safety measure: a review of the literature. Royal Automobile Clue of Victoria 2001/11/. Report No.: 01/03.

[16] Reichard AA, Marsh SM, Tonozzi TR (2017). Occupational injuries and exposures among emergency medical services workers. Prehosp Emerg Care.

[17] Usfa (2002) Safe operation of fire tankers. Emmitsburg, MD

[18] Abdelwanis N (2013) Characteristics and contributing factors of emergency vehicle crashes. 88

[19] Ahmed MA, Haynes K, Tofa M et al (2020) Duty or safety? Exploring emergency service personnel’s perceptions of risk and decision-making when driving through floodwater. Progress in Disaster Science 5:100068

[20] Bui DP, Hu C, Jung AM et al (2018) Driving behaviors associated with emergency service vehicle crashes in the U.S. fire service. Traffic Inj Prev 19(8):849–55 10.1080/15389588.2018.1508837 30605007

[21] Usfa (2003) Firefighter Fatalities in the United States in 2002. US Department of Homeland Security Federal Emergency Management Agency US Fire Administration

[22] Kahn CA, Pirrallo RG, Kuhn EM (2001). Characteristics of fatal ambulance crashes in the United States: an 11-year retrospective analysis. Prehosp Emerg Care.

[23] Knott M, Classen S, Krasniuk S et al (2020) Insufficient sleep and fitness to drive in shift workers: a systematic literature review. Accid Anal Prev 134:105234 10.1016/j.aap.2019.07.010 31443915

[24] Becker LR, Zaloshnja E, Levick N (2003). Relative risk of injury and death in ambulances and other emergency vehicles. Accid Anal Prev.

[25] Gormley M, Walsh T, Fuller R (2007). Risks in the driving of emergency service vehicles. Irish J Psychol.

[26] Hsiao H, Chang J, Simeonov P (2018). Preventing emergency vehicle crashes: status and challenges of human factors issues. Hum Factors.

[27] Missikpode C, Peek-Asa C, Young T, Hamann C (2018). Does crash risk increase when emergency vehicles are driving with lights and sirens?. Accid Anal Prev.

[28] James SM (2015) Distracted driving impairs police patrol officer driving performance. Policing: An Int J Police Strategies Manag 38(3):505–16

[29] Yager C, Dinakar S, Sanagaram M, Ferris T (2015) Emergency vehicle operator on-board device distractions 2015/02/19/

[30] Du B, Boileau M, Wierts K (2019). Existing science on human factors and ergonomics in the design of ambulances and EMS equipment. Prehosp Emerg Care.

[31] Slattery DE, Silver A (2009). The hazards of providing care in emergency vehicles: an opportunity for reform. Prehosp Emerg Care.

[32] Ray AF, Kupas DF (2005). Comparison of crashes involving ambulances with those of similar-sized vehicles. Prehosp Emerg Care.

